# Hybrid Domain Consistency Constraints-Based Deep Neural Network for Facial Expression Recognition

**DOI:** 10.3390/s23115201

**Published:** 2023-05-30

**Authors:** Xiaoliang Zhu, Junyi Sun, Gendong Liu, Chen Shen, Zhicheng Dai, Liang Zhao

**Affiliations:** National Engineering Research Center of Educational Big Data, Central China Normal University, Wuhan 430079, China; zhuxl@ccnu.edu.cn (X.Z.); sunjunyi@mails.ccnu.edu.cn (J.S.); gendong@mails.ccnu.edu.cn (G.L.); shenchen@mail.ccnu.edu.cn (C.S.); liang.zhao@ccnu.edu.cn (L.Z.)

**Keywords:** facial expression recognition, attention mechanism, attention consistency, JS divergence

## Abstract

Facial expression recognition (FER) has received increasing attention. However, multiple factors (e.g., uneven illumination, facial deflection, occlusion, and subjectivity of annotations in image datasets) probably reduce the performance of traditional FER methods. Thus, we propose a novel Hybrid Domain Consistency Network (HDCNet) based on a feature constraint method that combines both spatial domain consistency and channel domain consistency. Specifically, first, the proposed HDCNet mines the potential attention consistency feature expression (different from manual features, e.g., HOG and SIFT) as effective supervision information by comparing the original sample image with the augmented facial expression image. Second, HDCNet extracts facial expression-related features in the spatial and channel domains, and then it constrains the consistent expression of features through the mixed domain consistency loss function. In addition, the loss function based on the attention-consistency constraints does not require additional labels. Third, the network weights are learned to optimize the classification network through the loss function of the mixed domain consistency constraints. Finally, experiments conducted on the public RAF-DB and AffectNet benchmark datasets verify that the proposed HDCNet improved classification accuracy by 0.3–3.84% compared to the existing methods.

## 1. Introduction

Currently, with the rapid progress of deep learning algorithms and computer vision technologies, the accuracy of FER has improved gradually. As a result, FER is now being applied in various human-computer interaction systems, e.g., social robots, medical equipment, and fatigue driving monitoring. In the online learning field, facial expressions are important explicit state characteristics of the learners. Thus, determining how to improve the capability of learners’ emotion–state perception based on FER has attracted increasing attention in the online learning field [1]. However, when applied to online learning, the FER task faces the following problems [2]: (1) Images obtained under natural conditions meet low-quality problems. (2) FER must consider certain differences due to different usage scenarios. For example, in a natural scene with uncertain lighting and occlusion, it is necessary to adjust the parameters of the image adaptively and eliminate the influence of occlusion. In contrast, in a laboratory environment, better classification results can be obtained because there is less environmental interference. (3) Manual labeling processes are easily affected by the subjectivity of labelers, which results in noisy labels and affect classification accuracy. (4) Due to the interclass similarity and annotation ambiguity of facial expression data, the FER task is more challenging than traditional classification tasks.

Typically, traditional face recognition methods include four parts, i.e., the raw image input, data preprocessing, feature engineering, and expression classification [3]. Feature engineering, which is the most important process in traditional methods, needs to be extracted manually and input into the classifier for learning. The quality of feature extraction is closely related to the level of classification performance; thus, adaptability is weak, and the recognition accuracy is typically limited.

The end-to-end supervised deep learning method is the most common classification paradigm for FER, and its classification performance largely depends on a large amount of high-quality labeled data [4]. However, collecting large-scale datasets with highly accurate annotations is generally an expensive and time-consuming process, and training with an insufficient amount of data will result in poor generalization performance caused by overfitting. Thus, it is necessary to collect and clean data on a large scale or expand the number of samples using data augmentation techniques. In recent years, due to the powerful feature learning ability of deep learning methods, FER methods based on deep neural networks have made remarkable progress. For example, Wang et al. proposed SCN [5] to suppress the uncertainty of facial expression data to learn the robust features of FER. This network includes self-attention importance weighting, ranking regularization, and relabeling. The network uses a self-attention importance weighting module to learn the weight of each face image to capture the importance of the sample for training and for loss weighting. The ranking regularization module is employed to highlight certain samples and suppress uncertain samples, and the relabeling module attempts to identify mislabeled samples and modify their labels. Wang et al. proposed RAN to solve the FER problem under occlusion conditions and pose changes in natural scenes [6]. This network divides the input face image into several areas and inputs them to the backbone convolutional neural network (CNN) for feature extraction. It then uses a self-attention and relational attention module to summarize the facial region features in static images, and it introduces region-biased features to enhance the region weights for classification. Wen et al. proposed DAN to address the low recognition performance problem caused by the interclass similarity of facial expression images [7]. This network learns to maximize the class separability of the backbone facial expression features using a feature clustering network. Then, a multi-head cross-attention network captures multiple distinct attentions, and an attention fusion network punishes overlapping attentions and fuses the learned features. Zhang et al. proposed EAC to handle noisy labels from a feature-relearning perspective [8]. Their study exploits erasing attention consistency by designing an unbalanced framework to prevent the model from memorizing noisy labels. Liao et al. proposed a locally improved residual network attention model (RCL-Net). They introduced LBP features in the facial expression feature extraction stage to extract texture information on the expression image, emphasizing facial feature information and improving the model’s recognition accuracy [9]. Qiu et al. proposed a local sliding window attention network (SWA-Net) for FER. They proposed a sliding window strategy for feature-level cropping, which preserves the integrity of local features without requiring complex preprocessing. Their proposed local feature enhancement module mines fine-grained features with intra-class semantics through a multiscale, deep network. They introduced an adaptive local feature selection module to guide the model to find more of the essential local features [10].

Due to the limitation of network structure, the above methods cannot make full use of spatial information and channel information in facial images, and lack of attention consistency constraints, resulting in a certain limitation on the recognition accuracy of the model. Therefore, the proposed HDCNet firstly extracts facial expression-related features in the spatial and channel domains, and then it constrains the consistent expression of features through the mixed domain consistency loss function. Finally, the network weights are learned to optimize the classification network through the loss function of the mixed domain consistency constraints. Unlike EAC, which only constrains feature learning in the spatial domain, the proposed HDCNet method further strengthens the consistency of the channel representation probability distribution in the channel domain and further enhances the contribution of image label-related regions to FER. Our primary contributions are summarized as follows:

(1) A simple and effective mixed-domain consistency constraint is proposed. Here, by extracting facial expression features in the spatial and channel domains and constraining the consistent expression of features by designing a mixed-domain consistency loss function, state-of-the-art FER accuracy is obtained. (The best SOTA accuracy on RAF-DB and AffectNet datasets reach 90.35% and 60.40%, respectively).

(2) In the spatial domain, the network is constrained, and the loss function is designed based on the prior assumption of spatial attention consistency. In the channel domain, the loss function is constrained. The loss function is designed based on the consistency of the channel representation’s probability distribution before and after image transformation.

(3) Multiple evaluation and ablation experiments are conducted on multiple benchmarks to verify the effectiveness of mixed domain consistency (demonstrating that 0.39–3.84% and 0.13–1.23% improvement is achieved on RAF-DB and AffectNet, respectively).

The remainder of this paper is organized as follows. Section 2 introduces the research on the attention mechanism, the principle of attention consistency, and applications of class activation maps. Section 3 describes the proposed methods and modules, as well as the overall architecture and design of the loss function. In Section 4, we discuss the experimental process and analyze the experimental results. Finally, the paper is concluded in Section 5.

## 2. Related Works

In this section, we introduce the attention mechanism, attention consistency, JS divergence, and other technologies involved in the proposed HDCNet.

### 2.1. Attention Mechanism

The essence of the attention mechanism is a set of weight coefficients independently learned by the network and a dynamic weighting method to emphasize the areas of interest while suppressing irrelevant background areas. Attention mechanisms can be broadly classified into channel attention, spatial attention, hybrid attention, and self-attention [11].

Channel attention can strengthen important features and suppress unimportant features by modeling the correlation between feature maps of different channels and assigning different weight coefficients to each channel. For example, SENet adjusts the feature response between channels adaptively through feature recalibration [12], and SKNet was inspired by Inception-block and SE-block, and it considers multiscale feature representation by introducing multiple convolution kernel branches [13,14]. The attention to feature maps at different scales allows the network to focus more on important scale features. In addition, ECANet uses one-dimensional sparse convolution operations to optimize the “upgrade first and then reduce dimensionality” strategy adopted by SENet using two multilayer perceptrons to learn the correlations between different channels [15].

Spatial attention attempts to improve the feature expression of key areas. Essentially, it transforms the spatial information in the original image into another space using a spatial transformation module and retains key information. It generates a weight mask for each position and weights the output, thereby highlighting specific target regions of interest while attenuating irrelevant background regions. For example, CBAM connects a spatial attention module based on the original channel attention [16]. Generally, spatial attention ignores the information interaction between channels because it treats the features in each channel equally.

Self-attention is a variant of the attention mechanism. The purpose of self-attention is to reduce the dependence on external information and utilize the inherent information of the feature as much as possible to interact with attention. It first appeared in the transformer architecture proposed by Google. Later, He et al. applied self-attention to the CV field and proposed the Non-Local module, which models the global context through the self-attention mechanism and effectively captures long-distance feature dependencies. Generally, the process of acquiring attention is mapped into three vector branches through the original feature map, i.e., Q (query), K (key), and V (value). First, the correlation weight matrix coefficients of Q and K are calculated, and then the weight matrix is normalized using the SoftMax function. Finally, the weight coefficients are superimposed on V to model the global context information [17]. The dual attention mechanism proposed by DANet applies the No-local concept to the spatial and channel domains simultaneously. Respectively, it uses the spatial pixel points and channel features as Q vectors to realize context modeling [18].

### 2.2. Consistency of Attention

Generally, the rationality of class activation mapping (CAM) heatmaps can reflect the performance of CNN classifiers [19]. If the attention heatmap emphasizes the semantic regions related to the considered labels, then the CNN classifier will exhibit better classification performance. However, it is very difficult to label relevant regions accurately on a large number of training images, and there may be discrepancies between different annotators when labeling relevant regions. Thus, a straightforward method to improve the plausibility of the attentional heatmap is to provide explicit supervision of label-related regions during CNN training.

As discussed in the literature [4], in multi-label classification tasks, various techniques, e.g., data augmentation and image transformation, are employed to improve the performance of CNN classifiers. However, even when the training images are augmented by these transformations, current CNN classifiers cannot maintain the consistency of attention under many spatial transformations. In other words, the attention heatmap of the image before the data augmentation is inconsistent with the inverse transformation of the heatmap of the image after data augmentation. Thus, we designed an unsupervised loss function called “Attention Consistency Loss” by considering the consistency of visual attention under spatial transformation to realize better visual perception rationality and better image classification performance.

### 2.3. Class Activation Mapping

The CAM technique is used to generate a heatmap in order to highlight the contributing regions of discriminative classes in images in a CNN and can realize interpretable analysis and saliency analysis of the images. CAM algorithms are primarily divided into activation-based methods and gradient-based methods.

The activation value-based method obtains the heatmap of the corresponding category by weighting and summing the output feature maps of the convolutional layer. The original CAM algorithm uses the output of the global average pooling layer as the weight to perform a weighted summation of the feature map [19]. The ScoreCAM method introduces the attention mechanism and obtains the attention weight of each channel through repeated pooling and convolution operations, and then it performs a weighted summation of the feature maps [20]. The ssCAM method adds a spatial attention mechanism based on ScoreCAM and further improves the accuracy of attention by performing spatial attention transformation on the feature maps [21]. In addition, the AblationCAM method calculates the contribution of each channel to the final result by eliminating channels one by one to obtain the heatmap [22].

The gradient-based method primarily uses the gradient backpropagation method to calculate the importance score of each position according to the gradient information of the output feature map of the convolution layer to the target category to obtain a heatmap. The GradCAM method performs global average pooling on the gradient of the output feature map, obtains the weight coefficient, and performs weighted summation on the feature map [23]. The GradCAM++ method adds the calculation of second-order information based on GradCAM to obtain a more refined heatmap [24], and the LayerCAM method involves an element-based calculation process. For each element of each feature map, there is a corresponding weight coefficient that reflects the importance of the feature map more finely than previous methods [25].

The above CAM algorithms have achieved good performance in several tasks, e.g., image classification and target positioning; however, they also have their own limitations and deficiencies. First, the gradient-based methods have certain restrictions in terms of the order of the convolutional and pooling layers in the network; thus, they cannot be directly applied to all types of network structures. Second, the gradient-based methods are susceptible to background noise, which leads to a reduction in the accuracy of the heat maps. There are also some problems with the method based on the activation value. For example, for some unbalanced datasets, the training model will focus on categories with a large number of samples, which will affect the generalization ability of the model on the test data. In addition, these algorithms only focus on the target category. In other words, they do not consider the influence of other categories; thus, some important contextual information may be ignored.

## 3. Proposed Method

In this section, we describe the proposed HDCNet method in detail.

**Figure 1:**
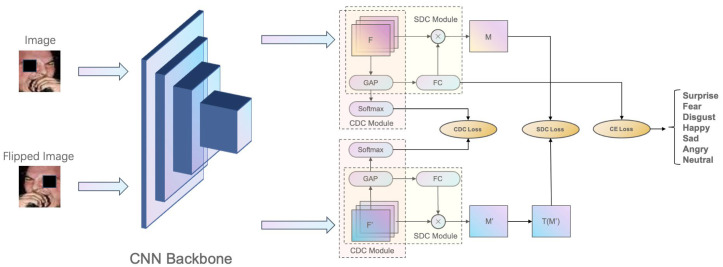
Overview of the proposed HDCNet. We note that M represents the heatmap corresponding to the original image, *M*′ corresponds to the heatmap after the horizontal flip operation, and T(·) represents the inverse transformation operation of the horizontal flip.

Most typical CNN architectures, e.g., the Inception [14] and VGGNet [26] networks, begin with convolutional layers, and then they perform global average pooling on the feature maps from the last convolutional layer. The pooled features are then input to the final fully connected (FC) layer for classification. The structure of the proposed HDCNet is shown in Figure 1. To learn high-quality features and realize better feature decoupling, our mixed domain consistency module is divided into two parts, i.e., the spatial domain consistency (SDC) constraint module and the channel domain consistency (CDC) constraint module, for short: SDC module and CDC module, respectively.

First, we perform data enhancement processing on a batch of images to obtain image *I* and its horizontally flipped image *I*′ satisfying the condition $I_{i,j,k}={I^{\prime }}_{i,j,w-k}$, where *i*, *j*, *k*, and *w* are the channel index, height index, width index, and width of the image, respectively. The images *I* and *I*′ are input to the backbone simultaneously, and after feature extraction, the feature maps *F* and *F*′ of the deep channel and low spatial resolution with basic category discrimination are obtained. In this study, we refer to the experimental results of EAC, where images are only processed with horizontal flipping and random erasing in the data augmentation stage.

The SDC module generates CAM heatmaps M and $M^{\prime }$ according to the feature map. Here, by minimizing the MSE difference between the attention maps, the network can learn the features related to the label from the spatial domain.

The CDC module generates probability distributions *P* and *Q* in the channel dimension according to the feature map. By minimizing the probability distribution difference, the network can stably learn the contribution distribution of each channel in the category discrimination, thereby reducing the label-independent features that are learned by the network.

**Figure 2:**
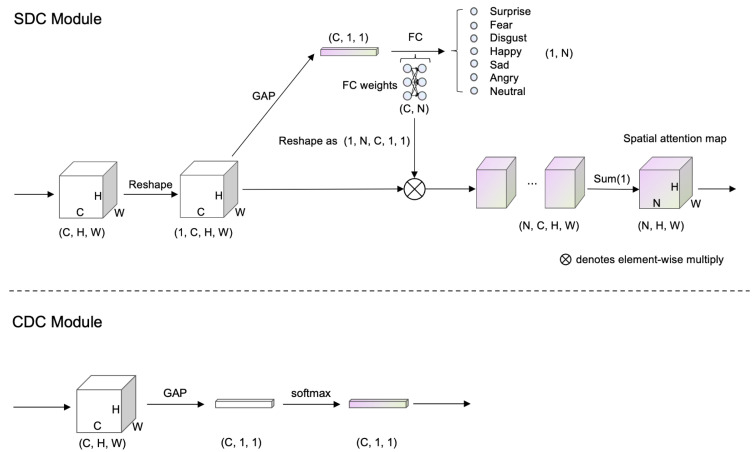
Overview of the SDC module and the CDC module. The figure shows how the dimensions of the image change as it passes through the SDC and CDC modules.

As shown in Figure 1 and Figure 2, we simultaneously input facial expression images processed with random erasing and horizontal flipping to the backbone CNN with ResNet50. One branch is the feature output from the original image, and the other branch is the feature output from the horizontally flipped image. The two output feature vectors are then processed by the CDC and SDC modules, respectively. Note that the CDC and SDC modules reuse the Global Average Pooling(GAP) layer. To be specific, the CDC module uses the SoftMax function to compress the features in the spatial dimension and infers the corresponding channel representation probability distribution. The SDC module integrates the global spatial information through the weighting of the FC layer to obtain the probability of the classification.

### 3.1. SDC Module

The GAP layer integrates the global spatial information. Here, the mean value after the GAP is weighted by the weight of the FC layer to obtain the probability of classification, and CAM weights the feature map before GAP to obtain the classification explanation. Typically, the rationality of the CAM heatmap can reflect the performance of the CNN classifier; thus, if the attention heatmap highlights regions that are semantically related to the considered label, the CNN will demonstrate better classification performance. The equation for GAP is as follows.

(1) $$y_{i}=\frac{1}{HW}\overset{H}{\underset{j=1}{\sum }}\overset{W}{\underset{k=1}{\sum }}x_{i,j,k}$$

here, H and W represent the height and width of the feature map, $x_{i,j,k}$ represents the value of the position of the feature map $\left(i,j,k\right)$, and *y_i_* represents the pixel mean corresponding to channel i.

**Figure 3:**
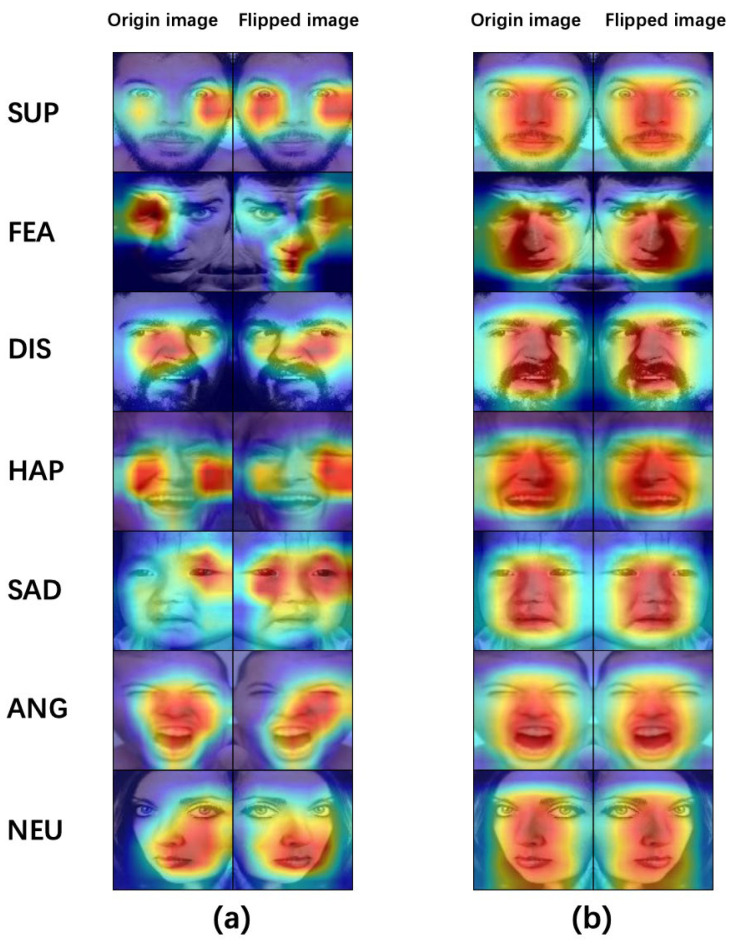
GradCAM heatmap of the original image and the corresponding horizontal flip image: (**a**) without SDC and CDC modules, and (**b**) with SDC and CDC modules.

As shown in Figure 3, the areas of interest in the image CAM heatmap before and after partial horizontal flipping do not overlap, which means that, although the semantic information before and after the image transformation has not changed, the network model is not consistent with the area of interest before and after the transformation. This demonstrates that the model does not fully focus on the label-related regions when performing the classification.

Table 1 are $F,F^{\prime }\in {\mathbb{R}}^{C\times H\times W}$, and the feature maps obtain the corresponding spatial average value after passing through GAP. Here, $W_{gap}\in {\mathbb{R}}^{C\times 1\times 1}$ is used as the weight of the feature map channel, and the weight of the FC layer of the final image classification is $W_{fc}\in {\mathbb{R}}^{C\times L}$. The shape of the feature map $F,F^{\prime }$ is changed to 1×C×H×W, and the shape of the FC weight $W_{fc}$ is changed to L×C×1×1, and multiply it linearly by channel. Combine the feature maps of each label and sum them along the channel dimension *C* to obtain the CAM heatmap $M\in {\mathbb{R}}^{L\times H\times W}$ corresponding to each label, formalized as follows.

(2) $$M_{l}\left(i,j\right)=\overset{C}{\underset{c=1}{\sum }}W\left(l,c\right)F_{c}\left(i,j\right)$$

here, L,C,H, and W are the number of classification tasks, the number of channels, height and width of the feature map, respectively. $M_{l}\left(i,j\right)$ indicates the attention heatmap of the label l at the spatial position $\left(i,j\right)$, $W\left(l,c\right)$ indicates the weight of the feature map channel *c* corresponding to the label *l*, and $F_{c}\left(i,j\right)$ represents the feature map of channel c from the last convolutional layer at the spatial position $\left(i,j\right)$.

We define the spatial domain consistency loss as the distance ($D_{sdc}$) between the attention heatmap before horizontal flipping and the attention heatmap after horizontal flipping. By optimizing the loss function, the spatial domain attention of the image is consistent before and after transformation, which can be expressed as follows.

(3) $$D_{sdc}=\frac{1}{LHW}\overset{L}{\underset{l=1}{\sum }}\|M_{l}-M_{l}^{\prime }\|{}_{2}$$

Equation (3) represents the spatial domain consistency distance for a single facial expression sample image, and the total spatial domain consistency loss is discussed in Section 4.3.

### 3.2. CDC Module

Given that each channel contains a specific feature response whose contribution to the final classification result is discriminative when exploring the consistency problem in the channel domain, we propose an a priori hypothesis: for a pair of feature maps $F,F^{\prime }\in {\mathbb{R}}^{C\times H\times W}$ output by the final convolutional layer, the CDC infers that the corresponding channel representation probability distributions *P* and *Q* are consistent.

(4) $$P=softmax\left(GAP\left(F\right)\right)$$

(5) $$Q=softmax\left(GAP\left(F^{\prime }\right)\right)$$

To measure the difference between two probability distributions, we first introduce the *KL* divergence to describe the difference from *P* to *Q*.

(6) $$KLD\left(P∥Q\right)=\sum p\left(x\right)log\frac{q\left(x\right)}{p\left(x\right)}$$

where (*x*) and *q*(*x*) denote the probabilities of *P* and *Q* on the *x*th event, respectively.

The higher the similarity between *P* and *Q* is, the smaller the *KL* divergence will be. Note that the difference from *Q* to *P* can be obtained in the same manner.

(7) $$KLD\left(Q∥P\right)=\sum q\left(x\right)\log\frac{p\left(x\right)}{q\left(x\right)}$$

However, due to the asymmetry of the *KL* divergence, the training efficiency may be reduced during the training process, and the convergence speed may be reduced. To solve this problem, we utilize the JS divergence to represent the difference between the two distributions.

(8) $$JSD\left(P∥Q\right)\&=\frac{1}{2}KLD\left(P∥M\right)+\frac{1}{2}KLD\left(Q∥M\right)$$

where $M=\frac{1}{2}\left(P+Q\right)$, *M* represents the intermediate distribution of *P* and *Q*. According to Equations (6)–(8), the simplified Equation (9) is obtained as follows.

(9) $$JSD\left(P∥Q\right)=\frac{1}{2}\sum p\left(x\right)log\left(\frac{p\left(x\right)}{p\left(x\right)+q\left(x\right)}\right)+\frac{1}{2}\sum q\left(x\right)log\left(\frac{q\left(x\right)}{p\left(x\right)+q\left(x\right)}\right)+log2$$

here, $P,Q\in {\mathbb{R}}^{C}$ represents the channel feature probability distribution of the feature $F,F^{\prime }$. Note that Equation (9) is the channel-domain consistency constraint loss for a single sample. Refer to Section 3.3 for the total channel-domain constraint loss.

### 3.3. Full Objective Function

We employ the cross entropy function as the classification loss function after the feature *f* of the GAP layer and the weight of the FC layer, $W_{fc}$, as follows.

(10) $${ℒ}_{cls}=-\frac{1}{N}\overset{N}{\underset{i=1}{\sum }}log\frac{e^{W_{fc}^{\left(y_{i}\right)}f_{i}}}{\sum_{j=1}^{L}e^{W_{fc}^{\left(y_{j}\right)}f_{i}}}$$

here, $W_{fc}^{\left(y_{i}\right)}$ represents the $y_{i}$-th weight of the FC layer, where $y_{i}$ is the label given by the *i*-th sample. In addition, $f_{i}$ represents the feature obtained by the feature *F* of the *i*-th sample through the GAP layer.

The loss function derived from Equation $\left(3\right)$ can be expressed as follows:

(11) $${ℒ}_{sdc}=\frac{1}{NLHW}\overset{N}{\underset{n=1}{\sum }}\overset{L}{\underset{l=1}{\sum }}\|M_{n,l}-M_{n,l}^{\prime }\|{}_{2}$$

here, $M_{n,l}$ represents the heatmap of the *l*-th category label of the *n*-th sample, and ${M^{\prime }}_{n,l}$ is the heatmap of the expression image corresponding to $M_{n,l}$, after flipping, where *N* is the sample size, *L* is the total number of classification task categories, and *H* and *W* are the height and width of the expression image, respectively.

The channel domain consistency constraint loss function derived from Equation $\left(9\right)$ can be expressed as follows.

(12) $${ℒ}_{cdc}=\frac{1}{N}\overset{N}{\underset{i=1}{\sum }}\left\{\frac{1}{2}\sum p_{i}\left(x\right)log\left(\frac{p_{i}\left(x\right)}{p_{i}\left(x\right)+q_{i}\left(x\right)}\right)+\frac{1}{2}\sum q_{i}\left(x\right)log\left(\frac{q_{i}\left(x\right)}{p_{i}\left(x\right)+q_{i}\left(x\right)}\right)+log2\right\}$$

Then, from Equations (10)–(12), we can clarify that the calculation method of the total objective function is expressed as follows.

$$L=L_{cls}+\lambda_{sdc}L_{sdc}+\lambda_{cdc}L_{cdc}$$

here, $L_{cls}$ represents the classification cross-entropy loss function, $L_{sdc}$ represents the spatial domain consistency constraint loss function, and $L_{cdc}$ represents the channel domain consistency constraint loss function. In addition, $\lambda_{sdc}$ and $\lambda_{cdc}$ are the hyperparameters of $L_{sdc}$ and $L_{cdc}$, respectively. For additional details, refer to Section 4.5.1, where the ablation experiments of $\lambda_{sdc}$ and $\lambda_{cdc}$ are discussed.

## 4. Experiment

### 4.1. Experimental Platform and Hyperparameter Settings

In this study, we used a PyTorch backend to implement the proposed HDCNet using a hardware platform with an Intel i7-10900K CPU, an NVIDIA RTX3080-10G GPU, and 64 GB of RAM. We used ResNet50 as the backbone network and fine-tuned the proposed HDCNet based on the pre-trained MS-Celeb-1M model. To facilitate a fair comparison, we cropped, aligned, and scaled the face images to 224 × 224 pixels. For data augmentation during training, we applied horizontal flipping and random erasing to the training images. During network training, the batch size was set to 64. The initial learning rate was set to $10^{-4}$, and the number of iterations was set to 60. In addition, the Adam optimizer was used to accelerate convergence. Here, a learning rate adjuster was utilized with a gamma ExponentialLR (a function of exponentially adjusted learning rate in PyTorch) value of 0.9 to reduce the learning rate in each round to prevent the accuracy rate from oscillating due to an excessively large learning rate during convergence.

### 4.2. Dataset

#### 4.2.1. RAF-DB

The RAF-DB is a real-world, large-scale facial expression dataset [27,28] commonly used in natural scene FER-related papers from the CVPR conference. This dataset contains approximately 30,000 facial images downloaded from the internet. The labels are based on crowdsourcing annotations, where each image was labeled by approximately 40 independent annotators who provided providing 7 basic and 11 composite sentiment labels. In this study, 15,339 images were used for expression classification (12,271 training set images and 3068 test set images). The images in this database exhibit significant differences in subjects’ age, gender and race, head posture, lighting conditions, occlusion (such as glasses, facial hair, or self-occlusion), and post-processing operations (such as various filters and special effects). RAF-DB has a lot of diversity, abundant and rich annotations.

#### 4.2.2. AffectNet

AffectNet is a large database of facial expressions in the wild, containing more than one million facial images collected from the internet by querying three major search engines using 1250 emotion-related keywords in six different languages [29]. About half of the retrieved images (~440 K) were manually annotated for the presence of seven discrete facial expressions (classification model) and the intensity of valence arousal (dimensional model).

### 4.3. Feature Visualization

**Figure 4:**
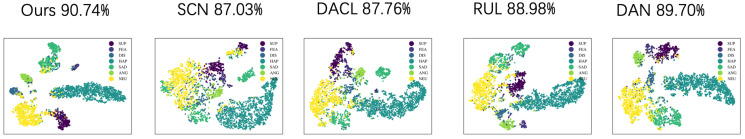
Cluster plots of T-SNE dimensionality reduction visualizations on the RAF-DB test set under different methods.

To evaluate the effectiveness of the proposed HDCNet, we used the PCA-initialized t-SNE algorithm [5,7,30,31] to visualize the distribution of features learned by the GAP layer on the test set. This method reduces the original high-dimensional features to two dimensions nonlinearly and uses the conditional probability of distance to represent the similarity between points. This distance is obtained by calculating the Euclidean distance. As shown in Figure 4, we compared the expression feature distributions of different models on the test set, and the results demonstrate that the proposed HDCNet outperformed the compared models [5,6,7,8] (their main ideas can be found in the Section 1). The expression feature distribution of the RAF-DB test set showed good intraclass compactness and interclass separability, i.e., the similarity between the same kinds of expression elements was high, the similarity between different types of expressions was low, and the classification interval was large. We believe that this is because HDCNet is forced to learn the features of the label-related regions and reduce the interaction with the features of the label-independent regions in the mixed domain consistency constraint, thereby improving the classification performance. Figure 4 shows the prediction results of the image before and after performing the flipping augmentation and the region of interest in the heatmap. As can be seen, due to the constraint effect of the loss function we designed, the network was consistent with the region of interest of the image before and after the horizontal flipping, which avoids the reduction in classification accuracy due to the semantic information change caused by the horizontal transformation of the image.

### 4.4. Gradient Class Activation Map Visualization

**Figure 5:**
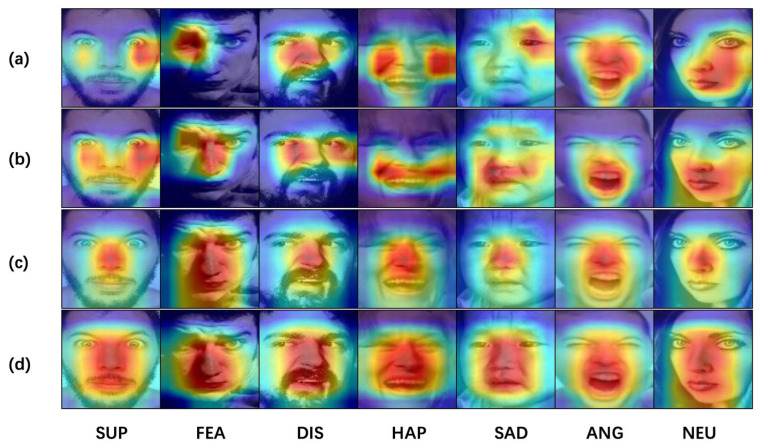
Proposed HDCNet’s CAM heatmap of different expressions on the RAF-DB test set. (**a**) without the SDC module and CDC module, (**b**) added the CDC module, (**c**) added the SDC module, and (**d**) both the SDC module and CDC modules are added. In Figure 5, the pictures in the “fear” category have uneven lighting, while the pictures in the “disgust” and “neutral” categories show significant changes in facial posture. The official website of the RAF-DB dataset introduces that its dataset has significant variability in head posture, lighting conditions, and occlusion. (http://www.whdeng.cn/raf/model1.html, accessed on 10 April 2023).

We used GradCAM to generate the heatmaps to visualize the contribution of the SDC and CDC modules to the FER task. First, we conducted experiments where the SDC and CDC modules were utilized independently, and then we conducted experiments in which both modules were used simultaneously. We then compared the heatmap results for these three cases. As shown in Figure 5: (a) without the SDC module and CDC module, (b) added the CDC module, (c) added the SDC module, and (d) both the SDC module and CDC modules are added.

When using only the CDC module, we found that the classification accuracy of the model increased by 1.64%, and the heatmap results demonstrate that the model’s attention to the mouth area increased significantly. When using only the SDC module, the classification accuracy of the model increased by 2.71%, and the heatmap results show that the model’s attention to the nasal area increased significantly. When the SDC and CDC modules were used simultaneously, the classification accuracy increased by 2.61%, and the heatmap results demonstrate that the models’ attention to the overall face area increased.

### 4.5. Ablation Study

#### 4.5.1. Weight Coefficient of the Loss Function

**Figure 6:**
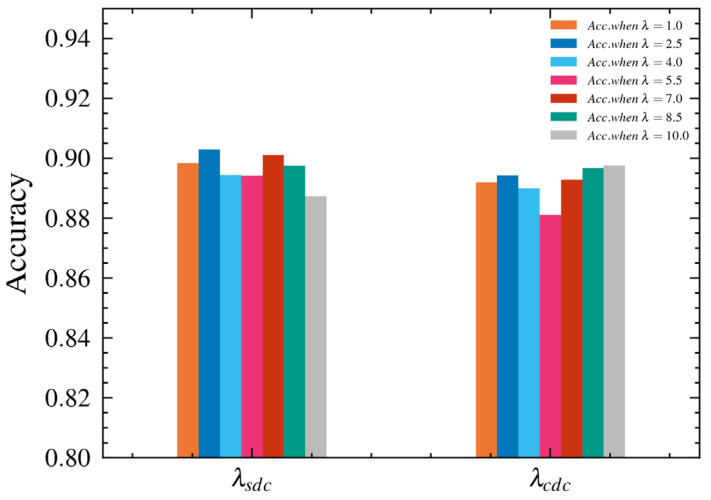
Performance comparison of the coefficients of the spatial domain consistency constraint loss function and the channel domain consistency constraint loss function on the RAF-DB dataset with an interval distance of 1.5 and a value range of 1.0–10.0.

To explore the influence of the SDC and CDC loss function weight coefficients on the performance of the proposed HDCNet, we conducted a series of experiments and selected different weight coefficients in the range of 1.0–10.0. Figure 6 shows the relationship between the classification accuracy of the proposed HDCNet on the RAF-DB dataset and different values for $\lambda_{sdc}$ and $\lambda_{cdc}$. The results show that when $\lambda_{sdc}$ = 2.5 and $\lambda_{cdc}$ = 10.0, the proposed HDCNet achieved the best performance. These experimental results verify the influence of the SDC and CDC loss function weight coefficients on the performance of HDCNet and provide an important reference for further optimization of the network.

#### 4.5.2. Ablation Experiments for the Effectiveness of SDC and CDC Modules

To verify the effectiveness of the SDC and CDC modules, we used ResNet50 as the benchmark comparison and conducted experiments on the RAF-DB dataset by adding the SDC module independently, adding the CDC module independently, and using SDC and CDC modules simultaneously. Without the SDC and CDC modules, the classification accuracy of ResNet50 on the RAF-DB dataset was 88.13%. When the CDC and SDC modules were used independently, the classification accuracy increased by 1.64% and 2.17%, respectively. When both CDC and SDC modules were used simultaneously, the classification accuracy increased by 2.61%. These results demonstrate that both the SDC and CDC modules have a positive contribution to the accuracy of the test set, and the effect of using both was better than using either module independently.

#### 4.5.3. CAM Algorithm Selection of SDC Module

Regarding the selection of the CAM algorithm in the SDC module, we considered the gradient-based GradCAM and LayerCAM algorithms. Although GradCAM is more general than CAM and does not require modification and retraining of the network, additional gradient information is required. The LayerCAM algorithm solves the problem of significant noise in GradCAM shallow feature maps but requires more computing resources. By comparing the results shown in Table 2, we observe that the original CAM algorithm obtained the best classification accuracy. The gradient-based CAM algorithm needs to backpropagate once more to obtain the gradient information, which incurs a huge computational overhead. However, the performance improvement is very limited. Thus, in Table 3, we quantify the average speed of each batch of the different CAM algorithms in the model processing. From the results, we selected the low-overhead original CAM algorithm.

### 4.6. Performance Comparison

Quantitative comparisons of performance on the RAF-DB dataset are shown in Table 1, Table 2 and Table 3. For a more convincing comparison, in Table 4, we compared the proposed method to the latest research in the FER field. As can be seen, the proposed method outperformed the two SOTA methods in classification accuracy, i.e., DAN [7] and EAC [8], by 1.04% and 0.39% on the RAF-DB, respectively. In particular, the fear, disgust, and sadness categories are 2.1%, 1.9%, and 5.1% higher than the EAC method, respectively.

**Figure 7:**
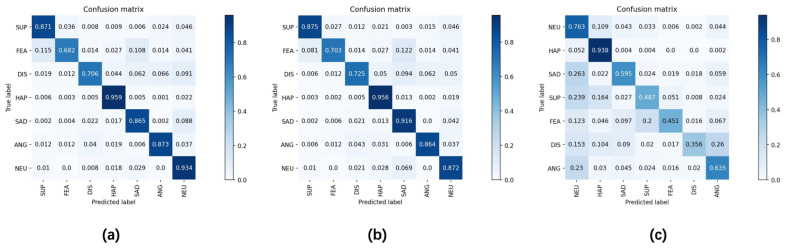
Confusion matrices of (**a**) EAC and (**b**) HDCNet methods on RAF-DB dataset. (**c**) HDCNet methods on AffectNet dataset.

To further measure the performance of the proposed method, we used a confusion matrix to evaluate the model’s prediction results. It lists the correspondence between the model’s prediction results and the real results, which can help us understand the model’s prediction performance, as well as the accuracy and error of the model on different categories. Here, each row represents the true label, each column represents the predicted label, and the diagonal represents the accuracy of the corresponding class. As shown in Figure 7, on the RAF-DB dataset, the recognition accuracy of happiness was the highest, and the recognition accuracies for both disgust and fear were the lowest. Note that the number of happiness samples was the largest in the experimental dataset, and the numbers of disgust and fear samples were small. As can be seen, disgust is easily confused with sadness, anger, and neutrality, and fear is easily confused with surprise, sadness, and neutrality. Compared with EAC, the proposed HDCNet improved the classification performance by 1.9% and 2.1% for both the disgust and fear categories with sparse samples. In addition, the classification accuracy of the proposed also increased by 5.1% in the sad category. These results demonstrate that the proposed HDCNet has a good effect in terms of handling sample imbalance, and it can improve the classification performance of the model. These results are in line with our expectations. Not only that, but we also made a comparison on the AffectNet dataset. Due to the long-tail distribution of the data, happiness and neutrality accounted for the majority. Other categories only occupied a small part, so the recognition accuracy of other categories was low.

## 5. Conclusions

In this paper, we have proposed the HDCNet to solve some difficult problems in the FER task, e.g., uneven illumination, large facial pose changes, occlusions, and low recognition accuracy caused by noisy labels in the target dataset. The proposed HDCNet consists of two parts, i.e., the SDC and CDC modules. The SDC module enhances the network to learn the label-related regions in the feature map by observing the areas of interest in the CAM heatmap before and after the feature map is flipped horizontally, thereby improving the model’s robustness. The CDC module assists the SDC module by minimizing the difference in the channel representation before and after the feature map is flipped horizontally such that the probability distribution of channel representation tends to be consistent. These two modules cooperate to improve both the accuracy and robustness of the model.

The proposed HDCNet was evaluated experimentally, and the experimental results on two benchmark datasets demonstrate that the proposed HDCNet achieved state-of-the-art performance, which highlights the effectiveness and practicality of the proposed method. We found that the proposed HDCNet can solve difficult problems in FER tasks; thus, it has broad applicability in practical applications.

Future work will explore new methods to measure channel domain consistency, improve the loss function, and make the model more robust to adapt to complex expression changes. We also plan to investigate the effectiveness of spatial domain consistency for feature layers at different depths and explore the effectiveness of using a mixed domain consistency method on lightweight models.

## Figures and Tables

**Table 1:** Results of ablation experiments on the effectiveness of SDC and CDC modules.

Methods	Accuracy (%)
-	88.13
+channel consistency	89.77 (+**1.64**)
+spatial consistency	90.30 (+**2.17**)
+channel consistency + spatial consistency	90.74 (+**2.61**)

**Table 2:** Comparison of classification accuracy of different CAM algorithms in the SDC module.

CAM-like Algorithm of SDC	Accuracy (%)
GradCAM	88.16
LayerCAM	87.71
CAM	90.30

**Table 3:** Performance overhead comparison of different CAM algorithms in the SDC module. A number in bold represents the best average processing speed result in the column.

CAM-like Algorithm of SDC	Average Processing Speed (Batch/s)
GradCAM	0.66
LayerCAM	0.59
CAM	**2.46**

**Table 4:** Performance comparison on RAF-DB dataset and AffectNet dataset. * denotes test with seven classes on the AffectNet dataset. ^†^ denotes that the model is pre-trained on MSCeleb. A number in bold represents the best accuracy result in the column.

RAF-DB		AffectNet	
Methods	Accuracy (%)	Methods	Accuracy (%)
SCN [5]	87.03	SCN [5]	60.23
RAN [6]	86.90	RAN [6]	59.50
RUL [31]	88.98	ESR-9 [32]	59.30
DAN [7]	89.70	EfficientFace [33]	59.89
EAC [8]	90.35	VGG-FACE [34]	60.40
Baseline1 (ResNet50)	83.46	Baseline1 (ResNet18)	53.81
Baseline2 ^†^ (ResNet50)	88.13	Baseline2 ^†^ (ResNet18)	56.97
HDCNet (Ours)	**90.74**	HDCNet * (Ours)	**60.53**

## Data Availability

Data underlying the results presented in this paper are available in RAF-DB [25,26], and AffectNet [27].

## References

[1] Li S., Deng W. (2022). Deep Facial expression recognition: A survey. IEEE Trans. Affect. Comput..

[2] Corneanu C.A., Simon M.O., Cohn J.F., Guerrero S.E. (2016). Survey on RGB, 3D, Thermal, and multimodal approaches for facial expression recognition: History, trends, and affect-related applications. IEEE Trans. Pattern Anal. Mach. Intell..

[3] Adjabi I., Ouahabi A., Benzaoui A., Taleb-Ahmed A. (2020). Past, present, and future of face recognition: A review. Electronics.

[4] Guo H., Zheng K., Fan X., Yu H., Wang S. (2019). Visual attention consistency under image transforms for multi-label image classification. 2019 IEEE/CVF Conference on Computer Vision and Pattern Recognition (CVPR).

[5] Wang K., Peng X., Yang J., Lu S., Qiao Y. (2020). Suppressing uncertainties for large-scale facial expression recognition. 2020 IEEE/CVF Conference on Computer Vision and Pattern Recognition (CVPR).

[6] Wang K., Peng X., Yang J., Meng D., Qiao Y. (2020). Region Attention networks for pose and occlusion robust facial expression recognition. IEEE Trans. Image Process..

[7] Wen Z., Lin W., Wang T., Xu G. (2022). Distract your attention: Multi-head cross attention network for facial expression recognition. arXiv.

[8] Zhang Y., Wang C., Ling X., Deng W. Learn from all: Erasing attention consistency for noisy label facial expression recognition. Proceedings of the 17th European Conference on Computer Vision (ECCV).

[9] Liao J., Lin Y., Ma T., He S., Liu X., He G. (2023). Facial expression recognition methods in the wild based on fusion feature of attention mechanism and LBP. Sensors.

[10] Qiu S., Zhao G., Li X., Wang X. (2023). Facial expression recognition using local sliding window attention. Sensors.

[11] Guo M.H., Xu T.X., Liu J.J., Liu Z.N., Jiang P.T., Mu T.J., Zhang S.H., Martin R.R., Cheng M.M., Hu S.M. (2022). Attention mechanisms in computer vision: A survey. Comput. Vis. Media.

[12] Hu J., Shen L., Sun G. (2020). Squeeze-and-excitation networks. IEEE Trans. Pattern Anal. Mach. Intell..

[13] Li X., Wang W., Hu X., Yang J. (2019). Selective kernel networks. 2019 IEEE/CVF Conference on Computer Vision and Pattern Recognition (CVPR).

[14] Szegedy C., Vanhoucke V., Ioffe S., Shlens J., Wojna Z. (2016). Rethinking the inception architecture for computer vision. 2016 IEEE Conference on Computer Vision and Pattern Recognition (CVPR).

[15] Wang Q., Wu B., Zhu P., Li P., Zuo W., Hu Q. (2020). ECA-Net: Efficient channel attention for deep convolutional neural networks. 2020 IEEE/CVF Conference on Computer Vision and Pattern Recognition (CVPR).

[16] Woo S., Park J., Lee J.Y., Kweon I.S. CBAM: Convolutional block attention module. Proceedings of the 15th European Conference on Computer Vision (ECCV).

[17] Wang X., Girshick R., Gupta A., He K. (2018). Non-local neural networks. 2018 IEEE/CVF Conference on Computer Vision and Pattern Recognition.

[18] Fu J., Liu J., Tian H.J., Li Y., Bao J., Fang Z.W., Lu H.Q. (2019). Dual Attention Network for Scene Segmentation. 2019 IEEE Conference on Computer Vision and Pattern Recognition (CVPR).

[19] Zhou B., Khosla A., Lapedriza A., Oliva A., Torralba A. (2016). Learning deep features for discriminative localization. 2016 IEEE Conference on Computer Vision and Pattern Recognition (CVPR).

[20] Wang H., Wang Z., Du M., Yang F., Zhang Z., Ding S., Mardziel P., Hu X. (2020). Score-CAM: Score-weighted visual explanations for convolutional neural networks. 2020 IEEE/CVF Conference on Computer Vision and Pattern Recognition Workshops (CVPRW).

[21] Wang H., Naidu R., Michael J., Kundu S.S. (2020). SS-CAM: Smoothed score-CAM for sharper visual feature localization. arXiv.

[22] Desai S., Ramaswamy H.G. (2020). Ablation-CAM: Visual explanations for deep convolutional network via gradient-free localization. 2020 IEEE Winter Conference on Applications of Computer Vision (WACV).

[23] Selvaraju R.R., Cogswell M., Das A., Vedantam R., Parikh D., Batra D. (2020). Grad-CAM: Visual explanations from deep networks via gradient-based localization. Int. J. Comput. Vis..

[24] Chattopadhyay A., Sarkar A., Howlader P., Balasubramanian V.N. Grad-CAM++: Improved visual explanations for deep convolutional networks. Proceedings of the 2018 IEEE Winter Conference on Applications of Computer Vision (WACV).

[25] Jiang P.T., Zhang C.B., Hou Q., Cheng M.M., Wei Y. (2021). LayerCAM: Exploring hierarchical class activation maps for localization. IEEE Trans. Image Process..

[26] Simonyan K., Zisserman A. Very deep convolutional networks for large-scale image recognition. Proceedings of the 3rd International Conference on Learning Representations (ICLR).

[27] Li S., Deng W., Du J. (2017). Reliable crowdsourcing and deep locality-preserving learning for expression recognition in the wild. 2017 IEEE Conference on Computer Vision and Pattern Recognition (CVPR).

[28] Li S., Deng W. (2018). Reliable crowdsourcing and deep locality-preserving learning for unconstrained facial expression recognition. IEEE Trans. Image Process..

[29] Mollahosseini A., Hasani B., Mahoor M.H. (2019). AffectNet: A Database for Facial Expression, Valence, and Arousal Computing in the Wild. IEEE Trans. Affect. Comput..

[30] Farzaneh A.H., Qi X. (2021). Facial expression recognition in the wild via deep attentive center loss. 2021 IEEE Winter Conference on Applications of Computer Vision (WACV).

[31] Zhang Y., Wang C., Deng W. Relative uncertainty learning for facial expression recognition. Proceedings of the 2021 NIPS Annual Conference on Neural Information Processing Systems (NeurIPS) NIPS.

[32] Siqueira H., Magg S., Wermter S. Efficient facial feature learning with wide ensemble-based convolutional neural net-works. Proceedings of the AAAI Thirty-Fourth Conference on Artificial Intelligence (AAAI).

[33] Zhao Z., Liu Q., Zhou F. Robust lightweight facial expression recognition network with label distribution training. Proceedings of the Thirty-Fifth AAAI Conference on Artificial Intelligence (AAAI).

[34] Kollias D., Cheng S., Ververas E., Kotsia I., Zafeiriou S. (2020). Deep neural network augmentation: Generating faces for affect analysis. Int. J. Comput. Vis..

